# Cardiomyogenic Differentiation Potential of Human Dilated Myocardium-Derived Mesenchymal Stem/Stromal Cells: The Impact of HDAC Inhibitor SAHA and Biomimetic Matrices

**DOI:** 10.3390/ijms222312702

**Published:** 2021-11-24

**Authors:** Rokas Miksiunas, Ruta Aldonyte, Agne Vailionyte, Tadas Jelinskas, Romuald Eimont, Gintare Stankeviciene, Vytautas Cepla, Ramunas Valiokas, Kestutis Rucinskas, Vilius Janusauskas, Siegfried Labeit, Daiva Bironaite

**Affiliations:** 1 Department of Regenerative Medicine, State Research Institute Centre for Innovative Medicine, Santariskiu Str. 5, LT-08460 Vilnius, Lithuania; rokas.miksiunas@gmail.com (R.M.); aldonyte.ruta@gmail.com (R.A.); 2 Department of Nanoengineering, Center for Physical Sciences and Technology, Savanoriu Ave. 231, LT-02300 Vilnius, Lithuania; agne@ferentis.eu (A.V.); tadas@ferentis.eu (T.J.); romuald@ferentis.eu (R.E.); gintare@ferentis.eu (G.S.); vytautas@ferentis.eu (V.C.); ramunas@ferentis.eu (R.V.); 3 Ferentis UAB, Savanoriu Ave. 231, LT-02300 Vilnius, Lithuania; 4 Clinic of Cardiovascular Diseases, Institute of Clinical Medicine, Faculty of Medicine, Vilnius University, M. K. Ciurlionio Str. 21/27, LT-03101 Vilnius, Lithuania; Kestutis.Rucinskas@santa.lt (K.R.); Vilius.Janusauskas@santa.lt (V.J.); 5 Medical Faculty Mannheim, University of Heidelberg, 68169 Mannheim, Germany; labeit@medma.de; 6 Myomedix GmbH, 69151 Neckargemünd, Germany

**Keywords:** cardiomyogenic differentiation, cardiac mesenchymal stromal cell, hydrogels, histone deacetylase inhibitors

## Abstract

Dilated cardiomyopathy (DCM) is the most common type of nonischemic cardiomyopathy characterized by left ventricular or biventricular dilation and impaired contraction leading to heart failure and even patients’ death. Therefore, it is important to search for new cardiac tissue regenerating tools. Human mesenchymal stem/stromal cells (hmMSCs) were isolated from post-surgery healthy and DCM myocardial biopsies and their differentiation to the cardiomyogenic direction has been investigated in vitro. Dilated hmMSCs were slightly bigger in size, grew slower, but had almost the same levels of MSC-typical surface markers as healthy hmMSCs. Histone deacetylase (HDAC) activity in dilated hmMSCs was 1.5-fold higher than in healthy ones, which was suppressed by class I and II HDAC inhibitor suberoylanilide hydroxamic acid (SAHA) showing activation of cardiomyogenic differentiation-related genes alpha-cardiac actin *(ACTC1*) and cardiac troponin T *(TNNT2*). Both types of hmMSCs cultivated on collagen I hydrogels with hyaluronic acid (HA) or 2-methacryloyloxyethyl phosphorylcholine (MPC) and exposed to SAHA significantly downregulated focal adhesion kinase (*PTK2*) and activated *ACTC1* and *TNNT2*. Longitudinal cultivation of dilated hmMSC also upregulated alpha-cardiac actin. Thus, HDAC inhibitor SAHA, in combination with collagen I-based hydrogels, can tilt the dilated myocardium hmMSC toward cardiomyogenic direction in vitro with further possible therapeutic application in vivo.

## 1. Introduction

Dilated cardiomyopathy (DCM) refers to heterogeneous myocardial disorders characterized by ventricle dilation and depressed myocardial performance [1]. It starts in the left ventricle and initiates chamber dilation, wall stretching, and thinning. Changed volume of the left ventricle further spreads to the right ventricle and then to the atria subsequently leading to the dilation of the whole heart [2]. Dilated cardiomyopathy and its further complications (heart failure, arrhythmias, thrombosis) are among the leading global causes of human mortality [3]. Current DCM treatment strategies are not always successful and often lead to heart transplantation. Therefore, new technologies modifying heart cells and/or extracellular environment to restore and/or regenerate damaged heart tissue remain relevant these days.

Studies investigating the effects of direct stem cell injection into heart muscle, epicardial patch or systemic cell deliver revealed embryonic, induced pluripotent, hematopoietic, adult mesenchymal stem/stromal (MSC), and cardiac progenitors being mostly employed for the cardiac regeneration studies both in vitro and in vivo [4,5,6]. However, stem cell transplantation-based DCM therapies have met several obstacles, such as cell-related tumorigenicity and immunogenicity, poor retention/engraftment, and insufficient tissue targeting [6]. The application of sophisticated tissue-engineered and cell-secreted products mimicking natural heart environment was also shown to improve survival of transplanted cells and subsequently human heart functioning [7,8,9]. However, the successful therapeutic engraftment of stem cells or extracellular environment-based components in the heart is still low due to the lack of more detail mechanistic investigations. Thus, new biosystems and/or biotechniques intracellularly and extracellularly stimulating cardiomyogenic differentiation processes are needed in order to improve failed heart functioning.

Histone deacetylases (HDACs) catalyze the removal of acetyl groups from lysine residues in the NH_2_ terminal tails of core histones, resulting in a more closed chromatin structure and repression of genes activity [10]. In addition to the histones, HDACs also control activity of various non-histone proteins such as tubulin, importin, Hsp90, and hypoxia factor-1 [11]. Altogether, 18 HDACs are recently known and grouped into the main four classes based on their structural and functional characteristics [12]. The class I HDACs (1, 2, 3, and 8) mainly control intracellular functions such as cell cycle, viability, proliferation and death, whereas class II HDACs (4, 5, 6, 7, 9, and 10) are more related to the tissue specific functions [12]. Class III HDACs include the Sirtuin (SIRT) family of seven proteins that are dependent on the NAD^+^ and do not contain zinc as do other HDACs [13]. Class IV HDAC include only HDAC11, which biological function has still not been fully investigated [14]. HDACs play a fundamental role in regulation of cell proliferation, survival, and differentiation, while impaired HDAC functions are widely involved in many pathophysiological processes such as cancer, neurodegeneration or inflammations-related disorders [15,16,17]. However, the role of HDACs and their inhibitors in DCM pathophysiology has not been fully investigated.

Cardiomyogenic differentiation in vitro can be induced by various factors inducing intracellular and extracellular changes. Accumulating data suggest that cells cultivated in conditions mimicking natural environment in vivo experience less stress and obtain more relevant phenotype [18]. It was also shown that adult tissue-derived MSC, when cultured for a long period on plastic surface, change cell shape and responses to the external stimuli [19]. Moreover, the plastic surface does not mimic the environment in which cells grow in vivo: the extracellular matrix (ECM) chemistry, cell-to-ECM contacts and other paracrine factors are changed. Extensive studies indicate that ECM components and integrin receptors-related signaling are very important regulators of myocardial hypertrophy, dilated cardiomyopathy, and even heart failure [20,21,22]. One of the major proteins involved in the ECM-cell signaling cascade is the non-receptor protein tyrosine kinase (FAK), which can be activated by growth factors or through the integrin-mediated cell attachment to the extracellular surfaces [23]. FAK activation has been shown also to be strongly involved in the regulation of myocyte hypertrophy model systems in vitro [24,25] and in vivo [26,27] and might be an important element regulating intracellular signaling systems and cardiac gene expression.

Since the main component of heart tissue ECM is collagen I (~80%), which has a tunable mechanical elasticity and ability to crosslink with various additives, it makes collagen I a very attractive biomaterial for cardiomyogenic differentiation studies [28]. Hyaluronic acid (HA) is another most abundant non-proteoglycan, a polysaccharide component of the heart ECM, which in combination with collagen I was successfully used for the rat heart tissue engineering purposes [29]. Recent study also showed that polymer 2-methacryloyloxyethyl phosphorylcholine (MPC), beside its structural similarity to the cell membrane phospholipids, has anti-inflammatory properties that were used to improve survival of long-term corneal implants [30]. Moreover, the corneal implants with MPC showed faster eye nerve regeneration and recovery than implants without MPC. Beside the chemical composition of the heart ECM mimicking biomatrices, their mechanical properties as well as surface topography can also regulate cell–cell, cell–ECM interactions changing an intracellular signaling and stimulating myogenic differentiation in vitro [31,32,33]. However, the impact of hybrid collagen I, HA, and MPC hydrogels and linear surface topography on the expression of cardiomyogenic differentiation-related genes in human dilated myocardium-derived hmMSC has not been investigated.

Therefore, the main goal of this study was to investigate abilities to tilt the dilated myocardium-derived hmMSC to the cardiomyogenic differentiation. We have also investigated the levels of MSC origin biomarkers, growth intensity, and HDAC activity in dilated myocardium derived-MSC comparing to the hmMSC derived from the normal functioning myocardium in vitro. The HDAC activity in dilated hmMSC was significantly higher compared to the healthy ones, which has been suppressed by class I and II HDAC inhibitor SAHA (also known as Vorinostat) subsequently activating expression of two main cardiomyogenic differentiation-related genes alpha cardiac actin (*ACTC1*) and cardiac troponin T (*TNNT2*). The impact of hybrid collagen I hydrogels with or without hyaluronic acid and polymer 2-methacryloyloxyethyl phosphorylcholine on the expression of *ACTC1* and *TNNT2* and possible role of FAK as well as longitudinal cell growth mode has been investigated. Data of this study show that HDAC inhibitor SAHA alone or in combination with hybrid collagen I-based hydrogels positively affect dilated hmMSC differentiation to cardiomyogenic direction in vitro.

## 2. Results

### 2.1. Cell Isolation and Morphology

**Figure 1:**
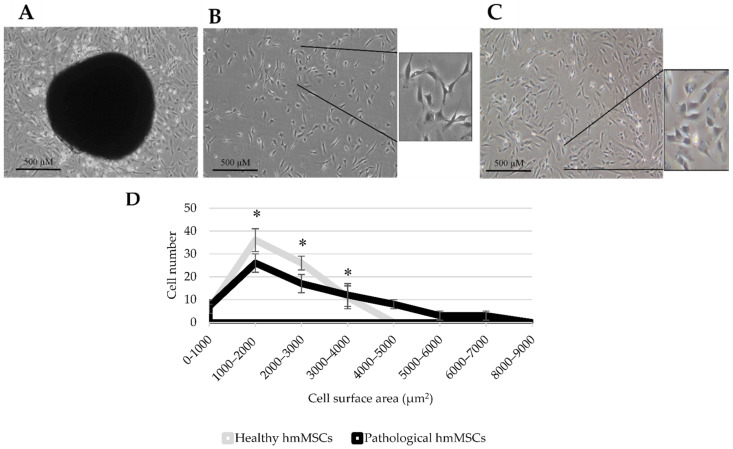
Morphology and size of healthy and pathological/dilated myocardium-derived hmMSC. (**A**) hmMSC outgrowth from human left ventricle myocardium biopsy material. (**B**) Healthy hmMSC in culture (2 weeks). (**C**) Pathological hmMSC in culture (2 weeks). (**D**) Cell size evaluation according to the cell attachment surface (µm^2^) using ImageJ software. Data are shown as mean value ± SD. * Data are significant at *p* ≤ 0.05 from not measuring not less than 60 cells (*n* = 60) of three patients from each group and comparing healthy and pathological cells. Statistical data were calculated using Excel software.

Human healthy and pathological (DCM myocardium-derived) hmMSC were isolated by explant outgrowth method and had typical stromal cell-like morphology (Figure 1A). hmMSC isolated from dilated human myocardium (Figure 1C) were bigger in shape and grew flatter on the plastic surface than hmMSC isolated from the healthy myocardium (Figure 1B). The size of healthy and dilated heart myocardium-derived hmMSC was calculated by evaluating cell attachment surface (µm^2^) (Figure 1D).

### 2.2. Characterization of Healthy and Dilated Myocardium-Derived hmMSC

Beside the morphology, we have also identified the MSC origin of healthy and pathological/dilated hmMSC by measuring levels of typical surface markers and differentiation potential to the main MSC directions (osteo, chondro, and adipo). The investigation of MSC typical biomarkers allows to evaluate the initial status of the healthy and dilated hmMSC and their further capabilities.

**Figure 2:**
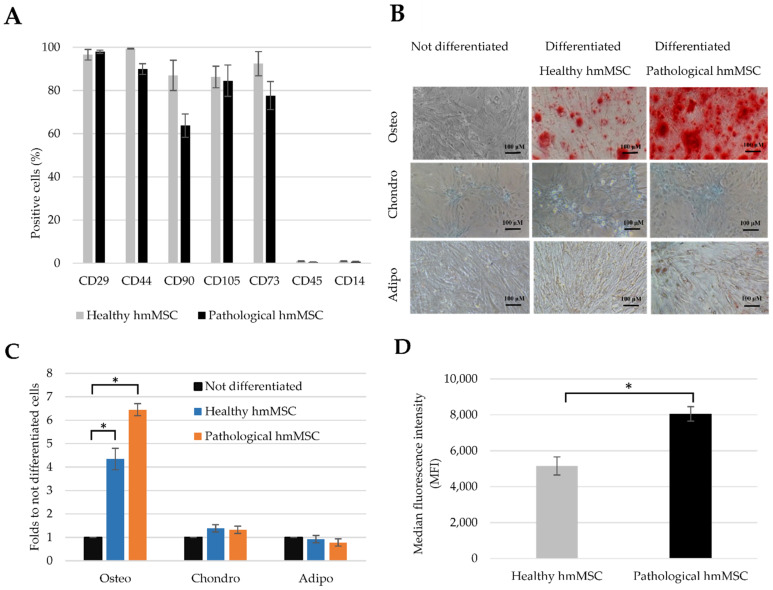
The identification of human healthy and pathological/dilated myocardium-derived hmMSC. (**A**) The levels of MSC-typical surface biomarkers on healthy and pathological hmMSC. (**B**) Qualitative evaluation of hmMSC differentiation towards osteo-, chondro-, and adipogenic directions (**C**) Spectrophotometric evaluation of osteo-, chondro-, and adipogenic differentiation. (**D**) Intracellular calcium level measured by Cal-520 dye. Data are shown as mean value ± SD. * Data are significant at *p* ≤ 0.05 level from three repeats (*n* = 3) of three patients from each group comparing osteo differentiated and not differentiated cells or intracellular calcium in healthy and pathological hmMSC using Excel software.

MSCs-typical surface markers were assessed by flow cytometer (Figure 2A). Healthy hmMSC were positive for CD29 (~98%), CD44 (~99%), CD90 (~87%), CD105 (~86%), and CD73 (~92%) and were nearly negative for CD45 (~1.1%), CD14 (~1.1%). Dilated myocardium-derived hmMSC (pathological) exhibited similar levels of surface markers: CD29 (~97%), CD44 (~90%) CD90 (~70%), CD105 (~85%), and CD73 (~78%).

The qualitative evaluation of differentiation potential of both types of the hmMSC to MSC-typical directions varied: cells showed osteo (strong), chondro (very weak), and adipo (almost not detected) (Figure 2B). Quantitative (spectrophotometric) evaluation of differentiation potential showed similar results (Figure 2C). Interestingly, both types of the hmMSC showed significantly increased differentiation to the osteo direction compared to the chondro and adipo (Figure 2C). The osteogenic differentiation was more prominent in the pathological hmMSC than in healthy MSC that could be related to the changed intracellular calcium level during DCM degeneration (Figure 2D). There is also a possibility that strong osteogenic differentiation of both types of the hmMSC is age-related sign: both cell types were isolated from elder men (55–65 years old) myocardiums that often show increased calcium deposits causing calcification and stiffening of the valve cusps in vivo. This part needs more detailed investigations.

### 2.3. Proliferation and Cardiomyogenic Differentiation of hmMSC on ECM Components-Precoated Plastic Surfaces

**Figure 3:**
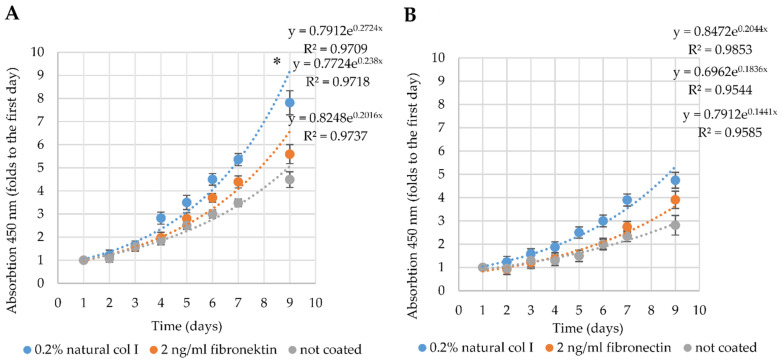
Proliferation of healthy and pathological/dilated myocardium-derived hmMSC on differently precoated and not precoated plastic surfaces. (**A**) Proliferation of healthy hmMSC. (**B**) Proliferation of pathological/dilated hmMSC. Both types of hmMSC were cultivated on surfaces precoated with 0.2% of natural collagen I, 2 ng/mL of fibronectin, and not precoated plastic surface for 9 days. Cell proliferation was measured by the CCK-8 kit. * Data are presented as mean of absorption (450 nm) ± SD and are significant at *p* ≤ 0.05 comparing cells grown on natural collagen I precoated plastic surface to not precoated or precoated with fibronectin. Not less than three repeats (*n* = 3) of three patients from each group were measured.

In order to understand, which type of hydrogel will be the best for the further cardiomyogenic differentiation studies, we investigated the proliferation of both types of the hmMSC on natural collagen I (0.2%) and fibronectin (2 ng/mL) precoated and not precoated plastic surfaces for nine days. Results indicate that healthy hmMSC proliferated faster than pathological hmMSC on all types of surfaces (Figure 3). The proliferation rate of both types of the cells was highest on natural collagen I precoated surface, compared to the plain plastic or fibronectin (Figure 3). Both types of the hmMSC showed exponential growth mode.

**Figure 4:**
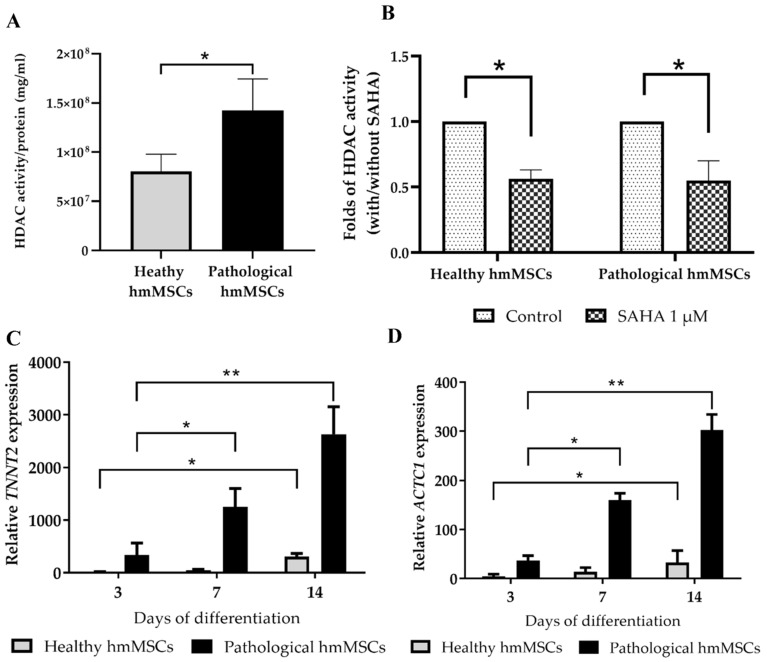
The effect of HDAC inhibitor SAHA on HDAC activity and cardiomyogenic differentiation of healthy and pathological/dilated myocardium-derived hmMSC. (**A**) HDAC activity in the healthy and pathological hmMSC. (**B**) The effect of SAHA on HDAC activity during 3 days of incubation. Expression of cardiomyogenic differentiation-specific genes: alpha-cardiac actin (*ACTC1*) (**C**) and cardiac troponin T (*TNNT2*). (**D**) The relative cardiac gene expression is shown as 2^−ΔCtx^2,000,000 and was normalized to the *ACTB* gene. Data are shown as mean ± SD and are significant at * *p* ≤ 0.05, ** *p* ≤ 0.01 levels from not less than three repeats (*n* = 3) of three patients from each group as calculated using the GraphPad Prism 6 software.

Further, we investigated the total HDAC activity (Figure 4A) and the effect of HDAC inhibitor SAHA on it (Figure 4B). The HDAC activity was significantly higher in the pathological cells (almost 50%) compared to the healthy hmMSC (Figure 4A), and was significantly suppressed by 1 µM SAHA during three days of incubation (Figure 4B). In parallel, we investigated the impact of 1 µM SAHA on the expression of most typical cardiomyogenic differentiation-related genes in vitro alpha-cardiac actin (*ACTC1*) (Figure 4C) and cardiac troponin T (*TNNT2*) (Figure 4D) in healthy and pathological hmMSC growing on plastic surface. The expression of *ACTC1* and *TNNT2* in both types of hmMSCs exposed to 1 µM SAHA for 14 days was upregulated (Figure 4C,D). HDAC inhibitor SAHA most significantly affected cardiac gene expression in pathological hmMSC. Data show that dilated myocardium-derived hmMSC still retained ability to differentiate to the cardio myogenic direction, which can be stimulated in vitro by epigenetic regulator SAHA.

### 2.4. The Attachment and Growth of Healthy and Dilated Myocardium-Derived hmMSC on Hybrid Collagen I Hydrogels

**Figure 5:**
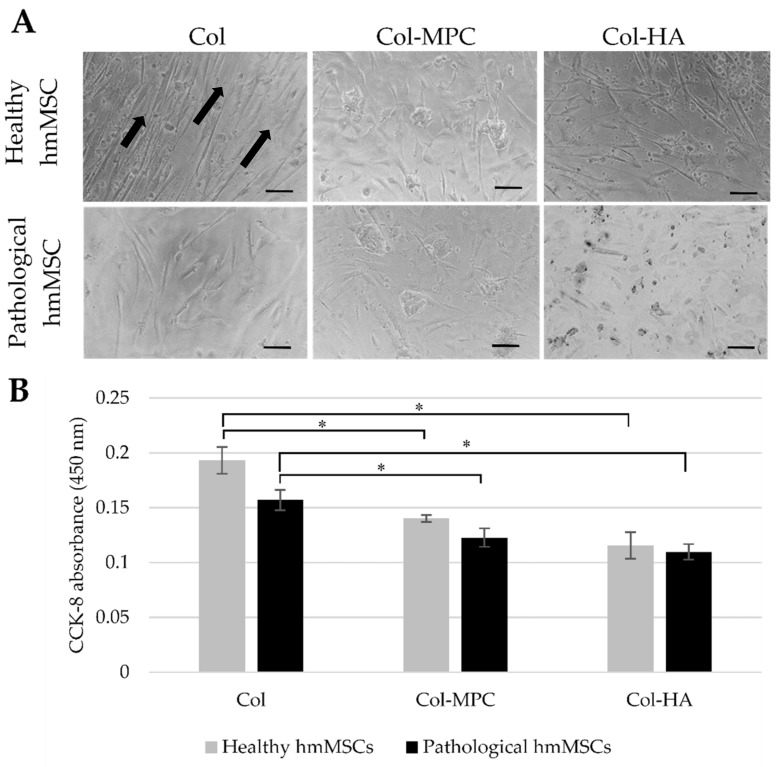
Attachment and survival of healthy and pathological/dilated myocardium-derived hmMSC on collagen I-based hydrogelss. (**A**) Healthy and pathological hmMSCs were allowed to attach to the collagen type I (Col) hydrogels, and collagen type I hydrogels with hyaluronic acid (Col-HA) and 2-methacryloyloxyethyl phosphorylcholine (Col-MPC) for 24 h. (**B**) Assessment of cell viability by CCK-8 absorption after 24 h in culture. * Significantly better attachment of hmMSC on Col hydrogels compared to the Col-MPC and Col-HA. Data are shown as absorption mean ± SD and are significant at *p* ≤ 0.05 from not less than tree repeats (*n* = 3) of two patients from each group. Scale bars are 100 µm. Arrows show linear growth mode of healthy hmMSC on Col hydrogel.

Since both types of hmMSC showed better proliferation on natural collagen I-precoated plastic surface compared to the fibronectin or plain plastic surface, we investigated hmMSC attachment and survival on collagen type I (Col)-based hydrogels supplemented with HA and MPC during 24 h of cultivation (Figure 5). The healthy and pathological hmMSCs showed better adherence to the Col hydrogels and aligned growth mode (Figure 5A, arrows) compared to the cells attached to the Col-MPC and Col-HA hydrogels (Figure 5). Overall, healthy hmMSC slightly better adhered to the all types of hybrid hydrogels compared to the pathological hmMSC (Figure 5A,B).

Further, we have investigated expression of cardiomyogenic differentiation genes *ATCT1* and *TNNT2* in healthy and dilated hmMSC cultured on hybrid hydrogels with and without HDAC inhibitor SAHA for 14 days. For the cardiomyogenic differentiation, both types of the hmMSC were cultured to complete confluence before adding SAHA.

### 2.5. The Differentiation to Cardiomyogenic Direction of Healthy and Pathological hmMSC on Hybrid Collagen Type I Hydrogels

**Figure 6:**
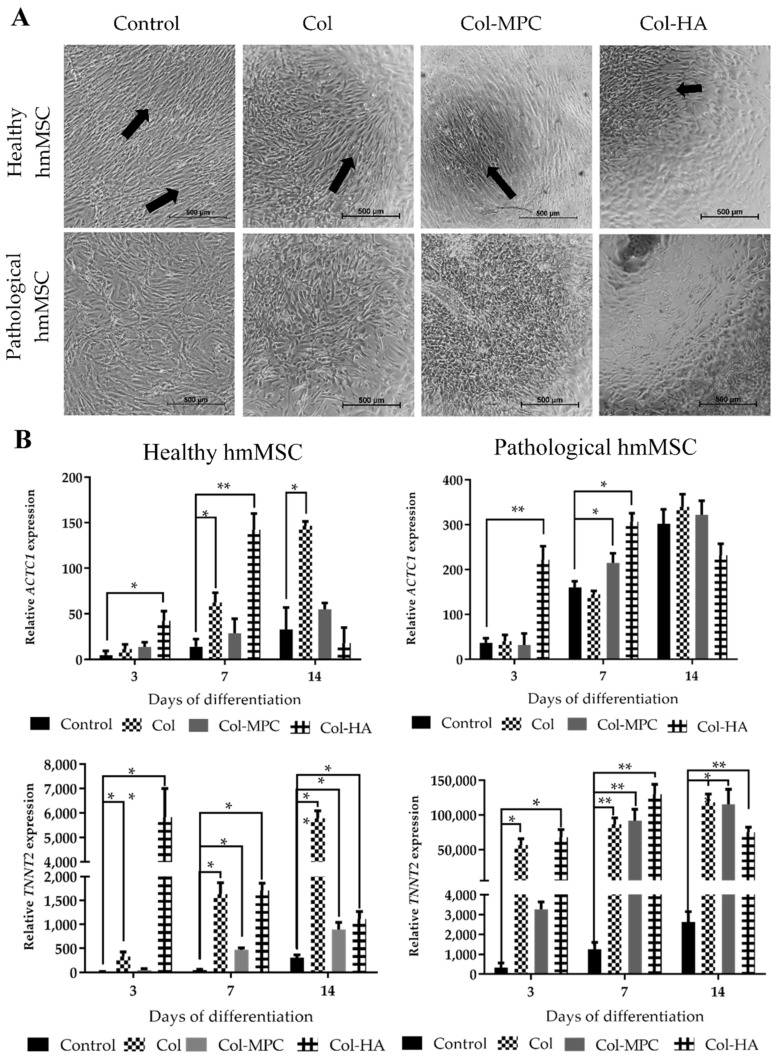
Expression of alpha-cardiac actin (*ACTC1)* and cardiac troponin T (*TNNT2*) genes in healthy and pathological/dilated myocardium-derived hmMSC during exposure to 1 µM SAHA for 3, 7, and 14 days. (**A**) Morphology of healthy and pathological hmMSC during 14 days of exposure to 1 µM SAHA on Col, Col-MPC, Col-HA hydrogels and plastic (Control). (**B**) Expression of *ACTC1* (*upper panels*) and *TNNT2* (*lower panels*) in healthy and pathological hmMSC grown on Col, Col-MPC, Col-HA hydrogels and plastic (Control). Relative cardiac gene expression is shown as 2^−ΔCtx^2,000,000 and was normalized to the *ACTB* gene. Data of 3 replicates (*n* = 3) of two patients in each group are shown with ±SD. Data are significant at * *p* < 0.05, ** *p* < 0.01 comparing expression of *ACTC1* and *TNNT2* cultivated on the hydrogels with the cells cultivated on plastic. The arrows indicate the longitudinal growth pattern of healthy hmMSC.

Since both types of hmMSC better attached and grew on Col hydrogel than on Col-MPC and Col-HA, we further investigated the impact of collagen I-based hydrogels on SAHA-stimulated cardiomyogenic differentiation of hmMSCs. In order to investigate the impact of hydrogels, healthy and pathological/dilated hmMSC were grown to monolayer on Col, Col-HA, and Col-MPC hydrogels and exposed to 1 µM SAHA for 3, 7, and 14 days (Figure 6). The differentiation of hmMSCs to cardiomyogenic direction was estimated by the expression of the *ACTC1* and *TNNT2* genes and compared to the cells cultivated on plastic.

The healthy hmMSC maintained more linear growth pattern on hybrid collagen I-based hydrogels compared to the pathological hmMSC during 14 days of cultivation (Figure 6A, arrows). All employed hydrogels showed different impact on *ACTC1* expression over the time: the highest *ACTC1* expression in both types of hmMSC cultivated on Col-HA was at the seventh day (142.5 ± 17.7 and 306.6 ± 18.8-fold, for the healthy and pathological cells, respectively), whereas hmMSC cultivated on Col and Col-MPC hydrogels most intensively expressed *ACTC1* at the 14th day (146.5 ± 4.8 and 55.1 ± 7.2-fold for the healthy, and 339.2 ± 28.2 and 322.1 ± 31.1-fold for the pathological cells, respectively) compared to the cells cultivated on plastic (Figure 6B, upper panels). The lowest *ACTC1* activation was in the cells cultivated on plastic (control).

A similar tendency was observed in *TNNT2* expression: the level of *TNNT2* in healthy and pathological hmMSC cultivated on Col-HA matrices was strongly upregulated at the third (5830.6 ± 1174.6 and 67812.3 ± 11048.2-fold, respectively) and seventh (1708.1 ± 152.7 and 130072.9 ± 14245.2-fold, respectively) day, whereas cultivated on Col and Col-MPC hydrogels was stably increasing up to the 14th day compared to the cells cultivated on plastic (Figure 6B, lower panels). The lowest stimulation of *TNNT2* expression, similar to *ACTC1,* was in the cells cultivated on plastic (Figure 6B, lower panels). In general, the activation of *ACTC1* and *TNNT2* was stronger in pathological hmMSCs, compared to the healthy ones, suggesting retained regeneration potential of dilated myocardium.

### 2.6. The Expression of Focal Adhesion Kinase (FAK) in Healthy and Pathological hmMSC Grown on Hybrid Collagen I Hydrogels

**Figure 7:**
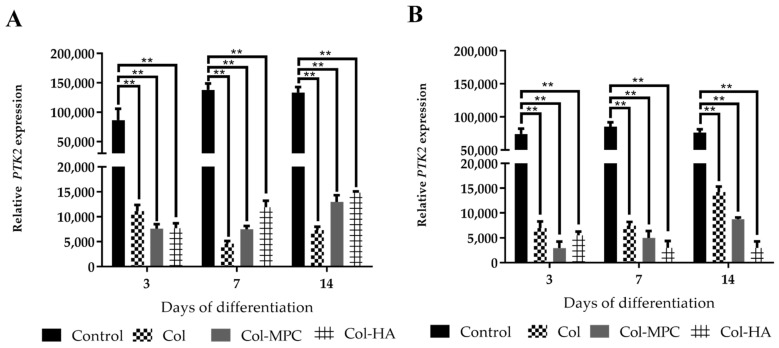
Expression of focal adhesion kinase gene (*PTK2)* in healthy and pathological/dilated myocardium-derived hmMSC cultivated on plastic and Col, Col-MPC and Col-HA hydrogels, and exposed to 1 µM SAHA for 3, 7, and 14 days. (**A**) Expression of focal adhesion kinase (*PTK2)* gene in the healthy hmMSC. (**B**) Expression of focal adhesion kinase gene *PTK2* in the pathological hmMSC. Relative *PTK2* gene expression is shown as 2^−ΔCtx^2,000,000 and was normalized to the *ACTB* gene. Data are shown as mean ± SD of three replicates (*n* = 3) from two patients of each group and are significant at ** *p* < 0.01 levels comparing expression of *PTK2* in the cells cultivated on the hydrogels with the cells cultivated on plastic.

In addition to *ACTC1* and *TNNT2*, the expression of FAK gene (*PTK2)* in the hmMSCs grown on the hybrid hydrogels has been estimated. FAK is a non-receptor protein tyrosine kinase participating in cell-ECM adherence signal transferring and stress responses. Data show that expression of FAK kinase gene *PTK2* was lower in both types of the hmMSC cultured on hybrid collagen I-based hydrogels compared to the plastic (Figure 7). In addition, the expression of *PTK2* in healthy hmMSC cultured on plastic was higher compared to the pathological hmMSC (Figure 7) suggesting healthy hmMSC being more sensitive to the adherence stress than pathological. Moreover, the lower expression of *PTK2* in both types of hmMSC grown on hydrogels can be explained by around 100-fold lower stiffness of all tested hydrogels: Col is ~140 kPa, Col-MPC is ~95, Col-HA is ~110 compared to the plastic (10,000 kPa) [31]. Reduced adherence-caused stress as well as hydrogel composition in the cells cultivated on hydrogels positively affected their cardiomyogenic differentiation-related gene expression.

### 2.7. The Impact of Longitudinal hmMSC Culturing on the Cardiomyogenic Differentiation-Related Proteins

**Figure 8:**
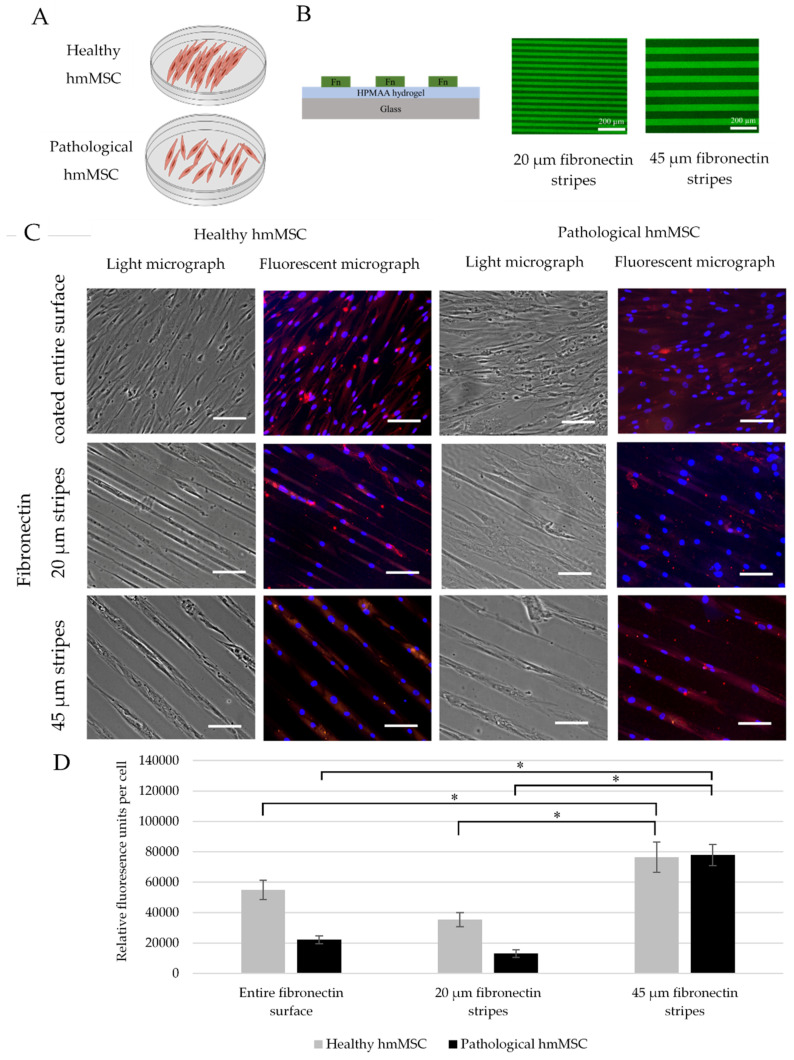
The level of alpha-cardiac actin in healthy and pathological/dilated myocardium-derived hmMSC cultured on uniformly-coated or linearly-printed fibronectin surfaces. (**A**) Schematic visualization of growth patterns of healthy and pathological hmMSC. (**B**) The schematic presentation of 20 ± 0.3 µm and 45 ± 1 µm fibronectin stripes. Fn—fibronectin, HPMAA—hydrophobic gel containing methyl methacrylic acid (MAA), 2-hydroxyethyl methacrylate (HEMA), and PEG methacrylate (PEG10MA). (**C**) Immunocytochemical micrographs of alpha-cardiac actin in healthy and pathological hmMSC. Upper panels—cells grown on uniformly fibronectin (2 ng/mL) coated surface, middle panel—on 20 ± 0.3 µm fibronectin-printed stripes, bottom panel—on 45 ± 1 µm µm fibronectin-printed stripes. Nuclei were stained with DAPI (blue). Scale is 100 µm. (**D**) Quantitative calculation of alpha-cardiac actin expression was evaluated by the fluorescence intensity (red) using ImageJ program and expressed as relative fluorescence units per cell. * Data are shown as mean of fluorescence intensity ± SD and are significant at *p* ≤ 0.05 compared fluorescence of the hmMSC on the 45 µm strips with the hmMSC fluorescence on 20 µm and entire fibronectin coating. Not less than 3 micrographs (*n* = 3) of the cells from three different patients of each group were measured.

In order to investigate the impact of linear pattern of hmMSC growth on the level of alpha-cardiac actin, the different width of fibronectin bioprinted stripes were used. The fibronectin lines were bioprinted on 1 cm diameter glass precoated with the low cell adherence material as shown in Figure 8 and described in the method part. The hmMSC cultivated on such biochips can attach only to the various topography of bioprinted fibronectin places.

Previous micrographs of this study (Figure 5, Figure 6 and Figure 8A, scheme) showed that healthy myocardium-derived hmMSCs grow in more neatly longitudinal manner than dilated myocardium-derived hmMSC. Both types of the hmMSC were cultivated on 20 ± 0.3 µm and 45 ± 1 µm wide fibronectin stripes-printed biochips (Figure 8B,C) for three days in IMDM medium without an additional stimuli and the level of alpha-cardiac actin was determined immunocytochemically (Figure 8C,D). In parallel, both types of hmMSC were cultivated on uniformly precoated fibronectin surface (Figure 8C, upper panels). The level of alpha-cardiac actin was strongly upregulated on 45 ± 1 µm fibronectin stripes compared to the 20 ± 0.3 µm stripes or entire coating (Figure 8D). Data show that artificially oriented longitudinal cell cultivation manner can increase the level of alpha-cardiac actin in both types of hmMSC without an additional stimulus. In addition, the expression of alpha-cardiac actin gene *ACTC1* in hmMSC on fibronectin lines was also evaluated by qPCR method using cell-to-Ct kit (Invitrogen™ TaqMan™ Gene Expression Cells-to-CT™ Kit), but the number of hmMSC was too low.

## 3. Discussion

It has long been thought that adult human heart is terminally differentiated organ without self-regenerating capabilities and only functions of cardiomyocytes (CM) were investigated [34]. However, it was shown that heart is composed of various types of the cells and even maintain slow regeneration potential [35,36]. It has been also shown that human cardiac regeneration may take place from the pre-existing CM [37,38], cardiospheres [39], or c-Kit^+^ cardiac progenitor cells residing in the heart tissue [40,41]. In addition, the MSC isolated from different types of adult tissues can have specific features: human fetal heart MSC was shown to have cardiac immunophenotyping and differentiation potential to CM, endothelial, or smooth muscle cells [42,43,44]. Human heart myocardium-derived MSCs can be used to investigate the human heart pathologies and/or possible ways of heart regeneration [45,46]. Other authors also suggested that adult human heart tissue-derived MSC have better cardio regenerating capabilities compared to the bone marrow or other types of adult MSC [47]. So far, it is a huge demand of cardiac tissue regenerating biomodels investigating the endogenous heart repair mechanisms, intrinsic and extrinsic signaling pathways, epigenetic, ECM-based and other molecular mechanisms [48]. In this study, the hmMSC were isolated from human healthy and DCM myocardium biopsies and their MSC origin [49], proliferation and ability to differentiate to cardiomyogenic direction combining intracellular (HDAC inhibitor SAHA) and extracellular biomatrices (hybrid collagen I-based hydrogels) has been investigated.

For a long time, HDACs were used as a promising tool for the development of new HDAC inhibitors, as potential anticancer drugs [50]. Recent studies suggested different members of class I and class II HDACs being important in cardiovascular physiology and pathophysiology: the interaction of class IIa HDACs with myocyte enhancer factor-2 (MEF-2) was shown to be an essential regulator of mouse cardiac hypertrophy [51,52]. The increased level of class I HDACs, particularly HDAC2, was directly related to the cardiac hypertrophy [53] or caused fatal cardiac arrhythmias [54]. Therefore, the investigation of HDACs activity and their inhibitors in heart diseases, particularly DCM, can be of promising therapeutic interests [55]. In this study, the dilated myocardium-derived hmMSC showed different properties compared to the healthy hmMSC: they were bigger in size, slower proliferated, showed more pronounced osteogenic differentiation than chondro and adipo, but had similar MSC-typical surface markers [56,57]. The strong osteogenic differentiation of both types of the hmMSC might be an age-related sign: both cell types were isolated from elder men (55–65 years old) myocardiums that often show increased calcium deposits causing calcification and stiffening of the valve cusps in vivo [45]. Moreover, the dilated hmMSC had significantly (1.5-fold) higher level of HDACs activity, which could negatively affect dilated myocardium. This finding encouraged to investigate the effects of HDAC inhibitors on dilated myocardium-derived hmMSC.

Data show, suberoylanilide hydroxamic acid SAHA (Vorinostat), a class I and II pan-histone deacetylase inhibitor, in addition to suppressing HDAC activity in pathological/dilated hmMSC, at the same time stimulating expression of cardiomyogenic differentiation-related genes. In previous studies, SAHA has been clinically approved for the treatment of cutaneous T-cell lymphoma (CTCL) and was shown to exert anticancer activities in various other types of tumors [58]. In addition, recent our publication showed SAHA suppressing *HDAC1* and *HDAC2* expression in dilated myocardium-derived MSC, improved mitochondrial activity and energetic status of human dilated myocardium-derived hmMSC [46]. Similar findings also confirmed HDAC1 and HDAC2 being the main regulators of histone acetylation [59]. Other HDACs can also negatively affect cardiac functioning: overexpression of *HDAC4* in mouse cardiomyocytes reduced functional recovery after ischemia/reperfusion injury, while excessive activity of HDAC6 in diabetic rats increased their vulnerability to ischemia/reperfusion injury [60,61]. On the other hand, the HDACs inhibitors slowed down fibrosis in hypertensive rats, improved mouse left ventricle end-diastolic pressure and cardiovascular functioning [62,63]. Despite the positive effect of HDACs inhibitors on animal hearts, their effect on human heart cells, particularly derived from the dilated myocardium, has not been investigated. Data of this study show HDAC inhibitor SAHA, in addition to the suppression of HDAC activity, stimulating expression of cardio myogenic differentiation-related genes *ACTC1* and *TNNT2*, particularly in dilated hmMSC. Further, the new type of hybrid collagen I-based hydrogels with HA and MPC have been developed to enhance SAHA effect on cardiomyogenic gene expression in hmMSC.

Collagen is the most abundant structural component of various tissues such as skin, tendon, lung, vasculature, and other in mammals [64]. In the heart, the extracellular collagen matrix supports cardiomyocytes and coronary microcirculation, ventricle diastolic functioning, vasculature system and transmits generated force to pump blood [65]. Fibrillar collagen type I and III are the major components of the heart extracellular matrix (ECM) constituting around 80% and 11%, respectively [66]. As natural collagen I is a mechanical-load flexible material, the main its role in various tissues is to maintain a tissue tensile and mechanical compression properties [67]. Injectable collagen hydrogels [68], as well as decellularized intact heart tissue ECM [69], have been used to stabilize ventricle, limit adverse remodeling, and improve cardiac functioning after myocardial infarction in animal modelling systems. However, as heart is a highly physically loaded organ, the hydrogels used for the mechanical studies in vitro or implanted in vivo should withstand high compression and/or compaction [70]. As biological function of collagen lies predominantly in its mechanical properties, its use for the heart regeneration purposes is the most reliable [28]. Therefore, due to the abundance of collagen I in the heart, biodegradability and mechanical-load elasticity, the hybrid type of collagen I-based scaffolds can be a promising therapeutic tool with the wide applicability in heart engineering field.

Beside the collagens, HA is the most abundant non-proteoglycan polysaccharide component of the heart ECM. In the heart, HA is involved in cardiac development at embryonic stage, healing processes, and also participates in pathological conditions such as atherosclerosis and myocardial infarction [71]. Since healthy and pathological cells had similarly high levels (99% and 90%, respectively) of CD44, a known HA receptor, data suggest that HA component in Col-HA hydrogel might be a dominating factor for the quick (3–7 days) activation of cardiac differentiation-related genes *ACTC1* and *TNNT2* in both types of the hmMSC, while on Col and Col-MPC it required longer (up to 14 days) of incubation time. There is also a possibility that HA, due to its hydrophilicity, could interrupt tight collagen packaging and improve cell binding to the hydrogel and subsequent quicker gene expression [72]. In addition, the combination of collagen I hydrogels with MPC, a cell membrane mimicking polymer [73], can regulate cell attachment [74], and was shown to be successfully used in corneal tissue regeneration [30,75]. Recently, MPC has been also shown to have anti-inflammatory feature that is important for the long-term tissue regeneration purposes [30]. Despite the fact that various composition of hydrogel biomatrices have been employed to mimic heart ECM [76], increase vascularization [77], deliver small molecules [78], or conveniently transplanted cells [79], the interest in complex cardiac tissue engineering techniques is constantly growing.

In addition, the positive impact of hydrogels on increased expression of cardiomyogenic differentiation genes *ACTC1* and *TNNT2* can be also related to the lower hydrogels stiffness: (Col = 140.28 ± 7.20 kPa, Col-MPC = 96.09 ± 13.19 and Col-HA = 113.02 ± 35.05 kPa compared to the plastic surface (10,000 kPa), while most tissues in our body are much softer (1–50 kPa) [31]. The softer hydrogel surfaces compared to the plastic decreased expression of cell adhesion stress-related focal adhesion kinase (*PTK2*) leading to the higher expression of cardiomyogenic differentiation genes. The stiffness of hydrogels used in this study differed little from each other, therefore, we assume that the composition of hydrogels also could have a significant impact on cardiac gene expression. Mechanical signals from the extracellular surfaces to the cells are usually transferred through the integrin-based adhesion and distinct molecules, such as FAK, which are particularly important for the further intracellular signal transfer [80]. It was shown that FAK, a non-receptor protein tyrosine kinase, also regulates cell adhesion-related responses such as cell proliferation, differentiation, and surface composition [23,31]. In addition, studies in vivo showed higher FAK level in the volume-overloaded human heart, which inhibition prevented load-induced cardiac hypertrophy in mice [81,82]. It was also shown that inhibition of FAK attenuated fibrosis in post-myocardial infarction mice model that could have a promising pharmaceutical strategy [83]. Data of this study also suggest that FAK expression in hmMSC grown on hydrogels compared to the hmMSC on plastic was downregulated that positively influenced cardiac gene expression.

Finally, cardiac regenerating strategies can be expanded via a hydrogel substrate surface topography and/or chemistry. Broad spectrum of ECM-based proteins or their derivative peptides can be incorporated into hydrogels or printed on their surface targeting special cells or tissues [84]. However, the linear cell growth pattern has been mostly investigated in the pro-myogenic techniques of skeletal myoblast such as murine myoblast cell line C2C12 and showing improved myotube formation by linear growth mode [85]. The oriented myoblasts growth also increased myotube fusion index and enhanced response to electrical pulse stimulation [86]. In another study, the linear nanofiber scaffolds promoted axial growth and enhanced cardiac differentiation of induced pluripotent stem cells (iPSC)-derived cardiomyocytes by stimulating cardiac troponin T expression [87]. Although axial growth on nanofiber scaffolds were shown to promote cardiac differentiation, there are no data showing the effect of aligned growth mode on human dilated myocardium-derived hmMSC. In this study, the specially bioengineered biochips with different breadth of fibronectin lines showed that both healthy and dilated hmMSC naturally stretching on 45 ± 1 µm strips can upregulate alpha-cardiac actin without any additional stimulus.

Though, data of this study show that dilated myocardium-derived hmMSC were bigger in size, slower proliferated, but had almost the same levels of MSC-typical cell surface markers compared to the healthy hmMSC. In addition, dilated myocardium-derived hmMSC had significantly higher HDAC level compared to the healthy myocardium cells, which can be suppressed by HDAC inhibitor SAHA leading to the increased expression of cardiomyogenic differentiation-related genes such as alpha cardiac actin and troponin T. Data also show that healthy and dilated myocardium-derived hmMSC cultured on collagen I-based hybrid hydrogels and exposed to SAHA more intensively expressed cardiomyogenic differentiation-related genes compared to the cell grown on plastic. The artificially oriented linear culturing of dilated hmMSC also stimulated the level of alpha-cardiac actin. Overall, the combination of intracellular stimuli such as epigenetic modulator SAHA with the extracellular (heart ECM-based hydrogels or other biomatrices) could be a suitable tool for targeted regulation of cardiomyogenic regeneration mechanisms and better understanding of DCM therapeutic and/or regenerating possibilities.

## 4. Materials and Methods

### 4.1. Isolation and Cultivation of hmMSC

Human healthy and dilated myocardium-derived primary hmMSC were isolated from biopsy material after obtaining patient’s (55–65 year-old men) written informed consent. The idiopathic dilated cardiomyopathy (NYHA group III) has been identified by the cardiologists according to the weak ventricle functioning (ejection fraction < 20%), diastolic diameter higher than 5.5 cm and other clinical parameters. Cell isolated from dilated myocardium were named as pathological or dilated hmMSC, while from the normally contracting myocardium, they were named healthy hmMSC. Not less than three patients’ samples of each group were used. Tissue fragments were washed in PBS (Sigma Aldrich, St. Louis, MO, USA), non-muscle tissue was removed, and fragments were cut into small pieces (2–3 mm in diameter). Biopsy material was pretreated with trypsin/EDTA (Thermo Fisher Scientific, Waltham, MA, USA), for 10 min, placed on fibronectin-coated (PeproTech, Rocky Hill, NJ, USA) 6-well surface and submerged in IMDM medium (Thermo Fisher Scientific, Waltham, MA, USA) containing 20% FBS (Thermo Fisher Scientific, Waltham, MA, USA) and antibiotics (Thermo Fisher Scientific, Waltham, MA, USA). Cell migration was allowed for 7–14 days until cells reached confluence of outgrowing. Cells were cultivated at 37 °C and 5% CO_2_ in a humidified atmosphere. The medium was changed twice a week. The confluent layer of cell outgrowth was trypsinized, washed with PBS and transferred to the 0.2% gelatine-coated cell culture flaks with IMDM, 10% of FBS, and antibiotics. hmMSC were used up to fifth passage number.

### 4.2. Evaluation of hmMSC Morphology

The size of healthy and pathological hmMSC was measured when cells reached a 50% confluence to better determine individual cell boundaries. Cell dimension was measured with the ImageJ software and analyzed using Microsoft Excel. The attachment area of individual hmMSC was marked and expressed as μm^2^. The data are presented as cell size mean ± SD evaluating not less than 60 cells from three patients’ biopsies of both types.

### 4.3. Identification of hmMSC Surface Markers

The MSC origin of human healthy and dilated myocardium-derived MSC (hmMSC) has been investigated as described elsewhere [43,46]. Briefly, cell growth media was aspired, washed with PBS and exposed to 0.25% trypsin-EDTA (Thermo Fisher Scientific, Waltham, MA, USA) for 1–2 min. After that, cells were suspended in 1% BSA (Sigma Aldrich, St. Louis, MO, USA) in PBS, counted, diluted to 0.5 × 10^6^ cells/mL, transferred into special flow cytometer tubes and incubated with specific fluorochromes-conjugated antibodies on ice for 30 min. Next, cells were washed with 1% BSA in PBS, centrifuged at 600 g for 5 min and resuspended in 1% BSA in PBS. Isotype controls were prepared. The cell samples were analyzed on BD FACSAriaTM IIU flow cytometer using the BD FACSDiva software (BD Biosciences, San Jose, CA, USA). Flow cytometry data were presented as a percent of cells having markers (for cell surface markers) and as fluorescence intensity (MFI) for other flow measurements.

The antibodies used to evaluate hmMSC surface markers were: CD29 (Integrin beta-1-ImmunoglobulinG1-Allophycocyanin (1A-219-T100, Exbio, Praha, Czech Republic)), CD44 (homing cell adhesion molecule-ImmunoglobulinG2b-Fluorescein isothiocyanate (555478, BD Biosciences, San Jose, CA, USA)), CD90 (thymocyte differentiation antigen 1-ImmunoglobulinG1-Fluorescein isothiocyanate (328108, BioLegend, San Diego, CA, USA)), CD105 (endoglin-ImmunoglobulinG1-Allophycocyanin (MHCD10505, Thermo Fisher Scientific, Waltham, MA, USA)), CD73 (ecto-5′-nucleotidase-ImmunoglobulinG1-Fluorescein isothiocyanate (561254, BD Biosciences, San Jose, CA, USA)), CD45 (protein tyrosine phosphatase, receptor type, C -ImmunoglobulinG2a-Fluorescein isothiocyanate (sc-70686, Santa Cruz Biotechnology, Dallas, TX, USA)), and CD14 (macrophage protein, which binds lipopolysaccharide-ImmunoglobulinG2a-Allophycocyanin (BioLegend, San Diego, CA, USA)).

### 4.4. Differentiation of hmMSC to Adipo-, Osteo-, and Chondrogenic Directions

Capabilities of hmMSC to differentiate toward at least one of MSC-typical directions such as adipo-, osteo-, or chondrogenic were investigated as described earlier with some modifications [88].

For the adipogenic differentiation, cells were grown to full confluence and the growth medium was changed to adipogenic differentiation medium (DMEM medium with 1 g/L glucose (Thermo Fisher Scientific, Waltham, MA, USA), 20% FBS (Biochrom GmbH, Berlin, Germany), 1 μM dexamethasone (Sigma Aldrich, St. Louis, MO, USA), 0.5 μM IBMX (MyBioSource, San Diego, CA, USA), 60 μM indomethacin (Sigma Aldrich, St. Louis, MO, USA)), which was changed twice a week. Differentiation was performed for 21 days. Adipogenic differentiation was identified by staining of fat (triglycerides and other lipids) droplets with Oil Red: the cells were fixed with glutaraldehyde and incubated with 12 mM Oil Red dye (Carl Roth GmbH, Karlsruhe, Germany). Red fat droplets were photographed under a light microscope, dye was extracted with 100% isopropanol (Eurochemicals, Vilnius, Lithuania) and the absorbance was measured at 520 nm. Cells directed to the adipogenic differentiation were compared to the control cells grown in normal growth medium.

For the osteogenic differentiation studies, cells were grown to 80% of confluence, the medium was changed to osteogenic differentiation medium (DMEM with 4.5 g glucose (Thermo Fisher Scientific, Waltham, MA, USA), 10% FBS (Biochrom GmbH, Berlin, Germany), 0.1 μM dexamethasone (Sigma Aldrich, St. Louis, MO, USA), 50 μg/mL L-ascorbic acid (Santa Cruz Biotechnology, Dallas, TX, USA), 10 mM β-glycerophosphate (Santa Cruz Biotechnology, Dallas, TX, USA), and 3 mM NaH_2_PO_4_ (Sigma Aldrich, St. Louis, MO, USA). Cell differentiation was performed for 21 days with medium changes twice a week. After 21 days of differentiation, the cells were fixed with cold ethanol, stained with 40 mM Alizarin Red (Carl Roth GmbH, Karlsruhe, Germany) (stains the calcified bodies) dye at room temperature (RT), photographed under a light microscope. The dye was extracted with 10% cetylpyridinium chloride (Sigma Aldrich, St. Louis, MO, USA) and absorbance was measured at 562 nm. Differentiated cells were compared to the cells grown in normal growth medium.

For the chondrogenic differentiation the cells were grown to the complete monolayer and growth medium was changed to the chondrogenic differentiation medium (DMEM media (Thermo Fisher Scientific, Waltham, MA, USA) with 0.1 µM dexamethasone (Sigma Aldrich, St. Louis, MO, USA), 0.17 mM ascorbic acid (Santa Cruz Biotechnology, Dallas, TX, USA), 0.35 mM proline (Carl Roth GmbH, Karlsruhe, Germany), 1x insulin-transferrin-selenium (Thermo Fisher Scientific, Waltham, MA, USA), and 10 ng/mL TGF-β3 (Thermo Fisher Scientific, Waltham, MA, USA) for 21 days with medium changed twice a week. After 21 days, cells were fixed with 95% methanol (Sigma Aldrich, St. Louis, MO, USA) and stained with 3% Alcian Blue dye (Carl Roth GmbH, Karlsruhe, Germany). Alcian blue dye acts as a large cationic molecule with several positive charges that bind to the negative polysaccharides on glycosaminoglycans and stain them blue. Alcian blue dye also stains acidic mucous mucins (glycoproteins that enter the mucous membranes of the glands). This paint belongs to the group of multivalent dyes that are soluble in water. The blue color is due to the presence of copper in the molecule. After incubation, the cells were photographed, incubated with DMSO (Sigma Aldrich, St. Louis, MO, USA), and absorbance was measured at 678 nm. The chondrogenic differentiation with TGF-β3 were compared with chondrogenic differentiation without TGF-β3.

### 4.5. Proliferation of Healthy and DCM Myocardium-Derived hmMSC

Proliferation was measured using the CCK-8 (Cell Counting Kit-8) kit. Cells with CCK-8 reagent were incubated at 37 °C for 3 h and absorption was measured spectrophotometrically at 450 nm. Tetrazolium salt-based assays provide a reliable and convenient platform to assess cell viability, proliferation and/or to test toxicity. Tetrazolium salt WST-8 (2-(2-methoxy-4-nitrophenyl)-3-(4-nitrophenyl)-5-(2,4-disulfophenyl)-2H-tetrazolium, monosodium salt) in cell counting kit (CCK-8) is reduced by cellular dehydrogenases to give an orange color product (formazan) that is soluble in cell culture medium and absorption can be measured at 450 nm. Viability was determined at indicated time points according to the manufacturers’ instructions (Dojindo, Kumamoto, JPN). Cell attachment to the gels 24 h after the seeding has been also measured by WST-8 reagent.

### 4.6. Evaluation of Calcium Concentration with Flow Cytometry

Cells (10^5^/well) were seeded in 6-well plate. Then, trypsinized cells were washed with PBS and incubated with 1 μM of calcium specific fluorescent dye Cal-520 (Interchim, Montlucon, France) at 37 °C for 30 min. After staining, cells were washed twice with PBS and 1% BSA and suspended in 1% BSA. Flow cytometry analysis has been performed with BD FACSAria II (BD Biosciences, San Jose, CA, USA).

### 4.7. Detection of HDAC Activity

The cells were washed twice with PBS, lifted with trypsin-EDTA solution, washed with PBS, and lysed with lysing buffer (50 mM Tris-HCl, pH 7.5, 5% glycerol, 0.3% Triton X-100). Protein concentration was determined with the Pierce™ Modified Lowry Protein Assay Kit. Cell samples were diluted with HDAC substrate buffer containing fluorescent HDAC substrate. HDAC activity was evaluated measuring fluorescence of universal HDAC substrate ((S)-tert-Butyl (6-acetamido-1-((4-methyl-2-oxo-2H-chromen-7-yl)amino)-1-oxohexan-2-yl)carbamate (BOC-Ac-Lys-AMC) diluted in HDAC substrate buffer (50 mM Tris- HCl, pH 8.1, 250 μM EDTA, 250 mM NaCl, 10% glycerol). The reaction was carried out at 30 °C for 30 min, where the HDACs cleave the acetyl group from the peptide substrate.

### 4.8. Preparation of Hydrogels

Collagen I-based hydrogels of 8.5% (*w*/*w*) (Col) were produced as previously described [89] using 4-(4,6-dimethoxy-1,3,5-triazin-2-yl)-4-methylmorpholinium chloride (DMTMM) (Merck KGaA, Darmstadt, Germany) as crosslinking agent. Shortly, 600 mg of collagen I (NMP collagen PS, Nippon Meatpackers, Ibaraki, Japan) aqueous solution of 12% (*w*/*w*) was mixed with 0.625 M 2-(N-morpholino)ethanesulfonic acid (MES) buffer (Merck KGaA, Darmstadt, Germany) within a glass syringe mixing system at RT. The calculated amount of a crosslinker was mixed in. The molar ratio of DMTMM to ε-amine groups of lysine in collagen molecule (Col-NH_2_) was 1:1. After thoroughly mixing, hydrogel solution was casted between two glass plates to form 500 µm thickness sheet. The hydrogel was then left to stay overnight in 100% humidity at RT.

To enhance stability against enzymatic digestion 6.5% (*w*/*w*) of collagen I hydrogels, the interpenetrating with 2-Methacryloyloxyethyl phosphorylcholine (MPC) polymeric network were fabricated as described earlier [90]. Briefly, 12% (*w*/*w*) of collagen solution was buffered with MES in a syringe mixing system, then MPC solution (Merck KGaA, Darmstadt, Germany) in MES was added, Col:MPC (*w*/*w*) ratio was 2:1. After mixing thoroughly, poly(ethylene glycol) diacrylate (PEGDA) (Merck KGaA, Darmstadt, Germany) was added (PEGDA:MPC (*w*/*w*) 1:3). Next, calculated volumes of 4% (*w*/*w*) ammonium persulfate (APS) (Merck KGaA, Darmstadt, Germany) (APS:MPC (*w*/*w*) 0.03:1) and 2% (*w*/*v*) N,N,N′,N′-tetramethylethane-1,2-diamine (TEMED) (Merck KGaA, Darmstadt, Germany) (APS:TEMED (*w*/*w*) 1:0.77) solutions in MES were mixed in. Then, a calculated amount of DMTMM (DMTMM:Col-NH_2_ (mol:mol) 1:1) was added. Hydrogel solution was mixed thoroughly and casted into 500 µm thickness sheets as described above. The final concentrations of Col and MPC in the hydrogel were 6.5% (*w*/*w*) and 3% (*w*/*w*), respectively.

For further enhancement of biointeractive properties of Col hydrogel, a new formulation of Col with hyaluronic acid (Merck KGaA, Darmstadt, Germany) was developed. The solution of 12% (*w*/*w*) Col was dispensed in a syringe mixing system as described above and a calculated amount of 1% (*v*/*v*) HA aqueous solution was mixed in. The final concentrations of Col and HA in the hydrogel were 8.5% (*w*/*w*) and 0.05% (*w*/*w*), respectively.

After crosslinking, mechanically robust and 500 µm thick Col, Col-HA and Col-MPC hydrogels sheets were trephined into 10 mm diameter cell culture substrate disks. Prior to use, hydrogel disks were kept refrigerated in PBS with traces of chloroform to maintain sterility. Tissue culture performance of hydrogel substrates was compared to conventional tissue culture plastic. All hydrogel parameters are shown in Supplementary Materials.

### 4.9. Preparation of Fibronectine Lines on Glass Chips

For the cell culture aligned growth experiments, in-house-made biochips were used. Biochips were prepared as described earlier [91]. Briefly, the biochips were 10 mm diameter and 130 µm thickness glass substrates coated with 34 ± 9 nm (in air) with 34 ± 9 nm (in air) poly(ethylene glycol) methacrylate hydrogel layer (PEGMA) (Merck KGaA, Darmstadt, Germany). Two different width fibronectins from Yo Proteins AB (Huddinge, Sweden) and bovine plasma fibronectin labelled with HiLyte™ Fluor 488 (FN-HiLyte™) (Cytoskeleton, Denver, CO, USA) lines 20 ± 0.3 µm and 45 ± 1 µm width with 20 ± 0.7 µm and 53 ± 1.2 µm spaces in between, respectively, were used. The fibronectin was microcontact printed on glass substrates (Merck KGaA, Darmstadt, Germany) poly(ethylene glycol) methacrylate hydrogel layer. The patterned surfaces were monitored using Olympus BX51 upright microscope (Olympus, Tokyo, Japan) equipped with 10xNA 0.3 air objective.

### 4.10. Cultivation of hmMSC on Hydrogels and Biochips

Prior to the cell cultivation, all hydrogels and biochips were washed (twice) with PBS in 37 °C and 5% CO_2_ incubator for 30 min. Next, all substrates were primed by submerging in cell culture medium for 30 min. The medium was then aspirated, substrates were left to dry at RT for about 10 min and cells were plated on the top of hydrogel and left to attach in 37 °C and 5% CO_2_ incubator for 24 h. Hydrogels were used to evaluate cell viability and cardiomyogenic differentiation for 14 days, while the expression of alpha cardiac actin was investigated on fibronectin stripes without any additional stimuli.

### 4.11. Cardiomyogenic Differentiation of Healthy and Dilated Myocardium-Derived hmMSC

For the cardiomyogenic differentiation, confluent hmMSC on hydrogels and on plastic were exposed to differentiation medium DMEM/F12 with 2% FBS, antibiotics and 1 µM of SAHA (Sigma Aldrich, St. Louis, MO, USA), and inhibitor of class I and II histone deacetylase (HDAC) inhibitor SAHA was added for 3, 7, and 14 days. Differentiation medium was changed every 2 days. Control cells were grown in DMEM/F12 medium (Thermo Fisher Scientific, Waltham, MA, USA) with 2% FBS and antibiotics without SAHA. Cardiomyogenic differentiation was evaluated by expression of alpha-cardiac actin and cardiac troponin T (cTnT) at gene and protein levels.

### 4.12. Gene Expression Levels

Cells grown on hydrogels and exposed to 1 µM SAHA for 14 days were washed, lysed in QIAzol^®^ reagent (Qiagen GmbH, Hilden, Germany) and centrifuged at 14,000× *g*, 4 °C for 10 min. For one time-point gene expression measurement cells were collected from ten hydrogels. Supernatant was mixed with 200 µL of chloroform (Sigma Aldrich, St. Louis, MO, USA), pipetted and incubated for 10 min at RT. Next, mix was centrifuged at 12,000× *g* for 15 min at 4 °C. Subsequently, RNA was collected from the upper phase, 0.5 mL of isopropanol was added and mix was incubated for 10 min at RT. After centrifugation at 12,000× *g*, 4 °C for 10 min RNA-containing pellets were collected and washed in ethanol (Sigma Aldrich, St. Louis, MO, USA) twice. Finally, RNA was resuspended in water and its concentration was assessed by SpectraDrop™ technology (Molecular Devices, San Jose, CA, USA). Complimentary DNA was synthesized by using the High Capacity cDNA Reverse Transcription Kit^®^ (Thermo Fisher Scientific, Waltham, MA, USA) according to the manufacturer’s suggestions. Produced cDNA was stored at −20 °C until used. PCR was run by using Maxima Probe qPCR Master Mix (2X), ROX Solution kit (Thermo Fisher Scientific, Waltham, MA, USA) in triplicates on the cycler AriaMx Real-Time PCR System^®^ (Agilent Technologies, Santa Clara, CA, USA). Temperature regimes were as follows: (1) denaturation and activation of Taq polymerase for 10 min at 95 °C, (2) denaturation for 15 s at 95 °C (40 cycles), (3) primer recognition and amplification for 60 s at 60 °C (40 cycles). The gene expression was calculated using 2^−∆Ctx^10,000,000 equation, where ΔCt is a subtraction of target gene Ct from housekeeping gene Ct, Ct is a threshold cycle. For the evaluation of the statistical significance 2^−∆Ctx^2,000,000 values were used. Gene expression was detected with these primers (Thermo Fisher Scientific, Waltham, MA, USA): *ACTC1* (Hs01109515_m1), *TNNT2* (Hs00943911_m1), and *PTK2* (Hs01056457_m1).

### 4.13. Immunofluorescence Assay

Cell cultures were fixed in 4% paraformaldehyde at RT for 15 min, washed with PBS, permeabilized with 0.1% Triton X-100 for 15 min and blocked with 1% BSA (Sigma Aldrich, St. Louis, MO, USA) at RT for 30 min as described in [92]. Primary antibodies to alpha-cardiac actin (GTX101876, GeneTex, Irvine, CA, USA) were added at dilution 1:50 and incubated at RT for 1 h. Washing steps with PBS and incubation with Alexa 549-conjugated secondary antibody (1:500) followed. Cells were visualized under fluorescent microscope Nikon Eclipse TE2000-U (Nikon, Tokyo, JPN). Cells were observed with the 4x objective after 14 days of differentiation on hydrogels and the 20 × objective was used in the experiments with fibronectin lines. All micrographs were done with Digital Sight DS-2MBWc camera attached to the Nikon Eclipse TE2000-U microscope. Red and UV filters were used to detect alpha-cardiac actin and DAPI staining, respectively. After staining, red (alpha-cardiac actin) and blue (DAPI) fluorescence was captured and analyzed with the ImageJ program. First, the average of cell surface area was determined by following formula:

Average cell surface area (µm^2^) = total surface covered by cells in one micrograph/number of cell nucleus stained with DAPI.

Second, we determined an average of fluorescence intensity of 1 µm^2^ background surface of fibronectin stripes. Thus, the average cell fluorescence was calculated by following formula:

Average of normalized fluorescence (relative fluorescence units per 1 µm^2^) = cell fluorescence in 1 µm^2^ surface − background fluorescence of fibronectin stripes in 1 µm^2^ surface.

Third, we evaluated relative fluorescence per cell area (µm^2^) by following formula:

Relative fluorescence per cell area = average of cell surface area (µm^2^) × average of normalized fluorescence (relative fluorescence units per 1 µm^2^).

### 4.14. Statistical Analysis

Statistical analysis was performed using Excel (Microsoft Corporation, Redmont, WA, USA) and the Graphpad Prism 6.01 (GraphPad Software, San Diego, CA, USA) software. The differences between measurements were analyzed for their statistical significance using the independent-samples Student two-sided *t*-test. Results from three patients’ cells of both types with not less than three-five repeats for each measurement were presented, unless indicated otherwise. Data were expressed as mean ± SD and were significant at * *p* ≤ 0.05 comparing with corresponding control samples.

### 4.15. Ethical Approval

The study was approved by the local Bioethics Committee (license No. 158200-14-741-257). All patients gave written informed consent to investigate heart samples. The investigation conforms to the principles outlined in the Declaration of Helsinki.

## 5. Conclusions

Overall, data from this study show that expression of cardiomyogenic differentiation-related genes such as cardiac actin and troponin T in dilated myocardium-derived hmMSC can be efficiently stimulated by combining epigenetic stimulus such as HDAC inhibitor SAHA and extracellular collagen I-based biomatrices or applying spatial/topographical clues. Data show that combining extra- and intracellular stimuli can increase expression of cardiomyogenic differentiation-related genes in human healthy and dilated myocardium-derived hmMSC in vitro. Findings of this study also show that collagen I-based hydrogel substrates better mimicked natural cardiac environment than plastic, decreased expression of cell adhesion stress-related focal adhesion kinase, and activated cardiac genes. In addition, the composition of collagen I-based hydrogels is not less important for the cardiac gene expression than stiffness and showed time-depended cardiac gene upregulation. Moreover, data from this study show that human dilated myocardium-derived hmMSC retained ability to upregulate cardiomyogenic differentiation-related genes, which can be purposefully stimulated by complex means in vitro. Further in vitro and in vivo studies are needed to assess the feasibility of this therapeutic approach.

## Data Availability

Data are contained within the article or Supplementary Material.

## Supplementary Materials

The following are available online at https://www.mdpi.com/article/10.3390/ijms222312702/s1.

## Abbreviations

ACTB	Actin beta
*ACTC1*	Alpha cardiac actin 1
CCK-8	Cell counting kit 8
APS	Ammonium persulfate
ECM	Extracellular matrix
DAPI	40,6-diamidino-2-phenylindole
DCM	Dilated cardiomyopathy
DMTMM	4-(4,6-dimethoxy-1,3,5-triazin-2-yl)-4-methylmorpholinium chloride
HA	Hyaluronic acid
HDAC	Histone deacetylases
hmMSC	Human myocardium-derived mesenchymal stem cell
MEF-2	Myocyte enhancer factor-2
MES	2-(N-morpholino)ethanesulfonic acid
MPC	2-methacryloyloxyethyl phosphorylcholine
MSC	Mesenchymal stem cells
PEGDA	poly(ethylene glycol) diacrylate
SAHA	Suberoylanilide hydroxamic acid
TEMED	N,N,N′,N′-tetramethylethane-1,2-diamine
*TNNT2*	Cardiac Muscle Troponin T
