# Supplementary material for: Cardiomyogenic Differentiation Potential of Human Dilated Myocardium-Derived Mesenchymal Stem/Stromal Cells: The Impact of HDAC Inhibitor SAHA and Biomimetic Matrices
Source: Int J Mol Sci. 2021 Nov 24;22(23):12702. doi: 10.3390/ijms222312702 (PMC8657551; doi:10.3390/ijms222312702)
Supplement: Supplementary file 1 [file ijms-22-12702-s001.zip › ijms-1461639-Supplementary.pdf]

Supplementary Methods

Characterization of hydrogels

The hydrogel samples were produced from the same raw materials following the same protocol as for the cell culture experiments presented in the main article. The total number of analyzed samples was nine.

The measurement of the total equilibrated water content (%) of the hydrogels was performed following the previously described protocol (Haagdorens et al., 2019, https://doi.org/10.1155/2019/7867613).

The hydrogel stiffness was measured using a Nanowizard 3 (JPK) atomic force microscope and a HQ:NSC36/tipless/no Al B probe (MicroMasch) modified with a 6,65 µm SiO_2 sphere. Each hydrogel sample was measured in 1× PBS solution in 3 different 50×50 µm zones with 1024 force-displacement curves recorded in each of them. The setpoint for the force-displacement curves was 120 nN and the approach was done at 50 µm/s. The Young's modulus was then calculated by fitting Hertz sphere to the plane model to approach a part of the force-displacement curves using the JPKSMP data processing software (version 6.1.120).

Supplementary Results

The highest elastic modulus (140,28 ± 7,20 kPa) was detected for the Col hydrogel containing 8.5 % (w/w) of chemically crosslinked collagen I. The collagen I hydrogel enriched with MPC and HA had lower Young's modulus values: 96,09 ± 13,19 kPa and 113,02 ± 35,05 kPa, respectively. The addition of 0.05 % (w/w) HA into 8.5 % (w/w) Col slightly increased swelling (visible from the increased water content compared to the Col hydrogel) and, therefore, could have reduced the stiffness of the hydrogel, whereas the decreased stiffness of Col-MPC hydrogel was likely related to the reduced collagen concentration in hydrogel from 8.5 % (w/w) to 6.5 % (w/w).

Table S1: Properties of the collagen I-based hydrogels

Hydrogel type	Col	Col-MPC	Col-HA
Visual inspection of transparency	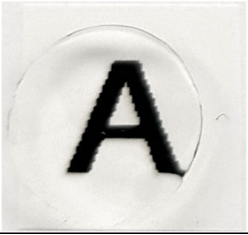	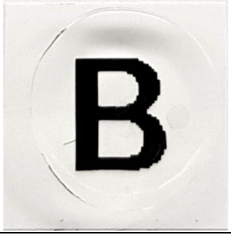	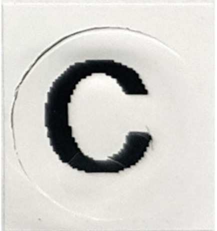
Measured water content, %	92,51 ± 0,12	94,71 ± 0,41	93,06 ± 0,12
Young modulus, kPa	140,28 ± 7,20	96,09 ± 13,19	113,02 ± 35,05
